# Pharmaceutical and Nutraceutical Potential Applications of *Sargassum fulvellum*

**DOI:** 10.1155/2020/2417410

**Published:** 2020-10-14

**Authors:** Jian Liu, Sibusiso Luthuli, Qifang Wu, Mingjiang Wu, Jong-il Choi, Haibin Tong

**Affiliations:** ^1^College of Life and Environmental Science, Wenzhou University, Wenzhou 325035, China; ^2^Department of Biotechnology and Bioengineering, Chonnam National University, Gwangju 500-757, Republic of Korea

## Abstract

*Sargassum fulvellum* is a brown seaweed of the Sargassaceae family which has been demonstrated to exhibit antipyretic, analgesic, antiedema, antimicrobial, antioxidant, antitumor, neuroprotective, anticoagulative, anti-inflammatory, and hepatoprotective activities. It has been widely used as a food additive and as a medicine in oriental medicine to treat lumps, dropsy, swelling, testicular pains, and urinary problems. *S. fulvellum* has been identified as a potential producer of a wide spectrum of natural compounds such as carotenoids, fucoidans, and phlorotannins, showing different biological activities in various industrial applications including pharmaceutical, nutraceutical, cosmeceutical, and functional food. However, the promising health effects associated with the extracts and compounds isolated from *S. fulvellum* have not been reviewed to date. The present review thus focuses on the biological activity of *S. fulvellum* as reported by previous publications, which include antioxidant, anticoagulant, anti-inflammatory, neuroprotective, immunomodulatory, antidiabetic, and anticancer effects. Thus, this review might serve to increase the utilization of this invaluable natural source as a potential component in pharmaceutical and nutraceutical applications.

## 1. Introduction

Seaweeds are used both as medicinal ingredients and as healthy foods in East-Asian countries such as China, Korea, and Japan [1]. Over the last years, seaweeds have been garnering considerable interest as sources of valuable functional metabolites [2]. The interest toward these biologically active components arises from their increasing potential as functional components in pharmaceutical, food, and cosmetic industries [3, 4].

*Sargassum fulvellum* (Turner) C. Agardh belongs to the brown seaweed (Phaeophyceae) order of the Sargassaceae family and is widely distributed throughout the Korean, Chinese, and Japanese coasts. It is also known as “Wu Lei Ma Wei Zao” (**无肋馬尾藻**) in Chinese, “Hondawara” (**馬尾藻**) in Japanese, and “Mojaban” (**모자반**) in Korean. This seaweed is regarded as an edible seaweed, and its dry material contains high contents of carbohydrates (30–60%), such as cellulose, fucoidan, laminaran, alginic acid, protein (15.8%), ash (27.5%), and relatively low amounts of fat (5%) [5, 6]. People living in Jeju Island (Republic of Korea) use this seaweed to prepare salads, soups (Mom-Guk), or side dishes [7–9]. In addition to its nutritional value, traditional medicine recommends the use of *S. fulvellum* to treat diseases such as a lumps, swelling, testicular pains, and urinary tract infections [10–12]. Previous studies have reported that *S. fulvellum* contains many bioactive molecules, such as phlorotannins, grasshopper ketone, fucoidan, and polysaccharides. *S. fulvellum* extracts or isolated pure components have been studied for years for analyzing their diverse pharmacological effects such as antioxidant, anticancer, anti-inflammatory, antibacterial, and anticoagulant activities [11, 13–15].

Since 2000, more than 40 papers have been published on the bioactive properties of *S. fulvellum*. However, there is still insufficient systemic data on its chemical constituents and pharmacological effects. This paper summarizes the research conducted over the past decades, on the chemical components of *S. fulvellum* and their biological activities. Nowadays, the therapeutic effects of this seaweed are scientifically credible and have been partially explained by *in vivo* and *in vitro* assays. Hence, our review summarized the literature on the biological characterization and pharmacological bioactivities of *S. fulvellum*, with an emphasis on its functional properties, which could provide a comprehensive insight into the chemical structures and biological activities of its active compounds and could also provide information that would lead to the development of more applications based on *S. fulvellum.*

## 2. Search Strategy, Data Collection, Evaluation, and Screening

In the current review, the relevant information on *S. fulvellum* was gathered from worldwide accepted scientific and academic databases via an electronic search such as Google Scholar, Web of Science, ScienceDirect, PubMed, Wiley Online Library, and Scopus. The following search terms were used as keywords and Boolean operator: “*Sargassum fulvellum*” + “extracts/compounds” or “polysaccharide” or “fucoidan” or “characterization” or “purification”; “*Sargassum fulvellum*” + “anticancer/antitumor” or “antioxidant” or “anti-inflammatory” or “immunomodulatory” or “anticoagulative” or “neuroprotective” or “hepatoprotective”. The review accessed and summarized the literature published until March 2020. No restrictions to any specific languages were made. All studies that met title and abstract criteria were selected for full-text review. A total of 84 research and review articles were reviewed for all mentioned databases; and only 46 potential research and review articles were screened out containing the maximum relevant information under the umbrella of different keywords. Afterwards, the authors carefully studied the selected articles in terms of content to write this review.

## 3. Important Biologically Active Components of *S. fulvellum*

**Figure 1:**
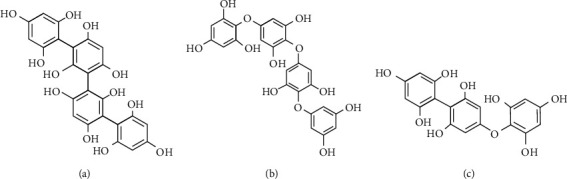
Example structures of the primary types of phlorotannins: fucols (a); phlorethols (b); fucophlorethols (c).

**Figure 2:**
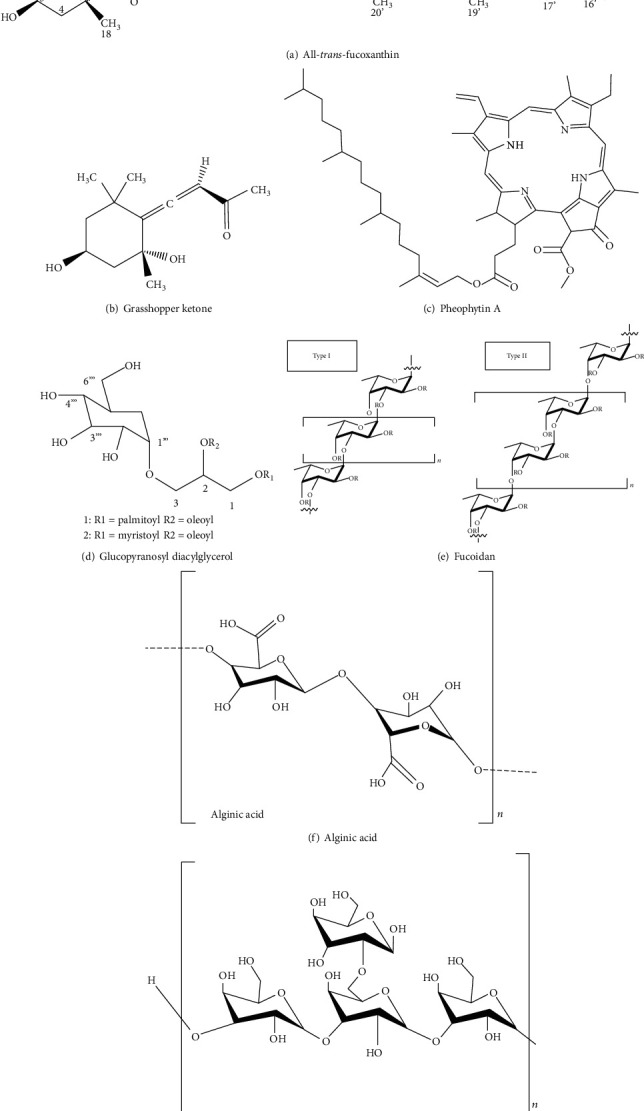
Structures of bioactive compounds isolated from *S. fulvellum*.

Marine organisms are rich sources of structurally diverse bioactive compounds, and their importance as sources of novel bioactive substances is growing rapidly [16]. *S. fulvellum* is a source of many health-promoting components, mainly phlorotannins, fucoxanthin, and polysaccharides. Surprisingly, phlorotannins have been found only in marine brown algae, which protect them from natural enemies by acting as herbivore deterrents, digestive inhibitors, ultraviolet (UV) screens, and antibacterial agents [17, 18]. Phlorotannins are structurally less complex than terrestrial tannins, as they comprise chains of 1,3,5-trihydroxybenzene formed via the acetate-malonate pathway [19, 20]. The phlorotannins can be categorised into three primary types according to the criteria of interphloroglucinol linkages: (i) fucols, (ii) phlorethols, and (iii) fucophlorethols (Figure 1) [21, 22]. Fucoxanthin (Figure 2(a)) is a well-known xanthophyll pigment, responsible for the brown color of seaweeds; it accounts for around 10% of the total natural seaweed carotenoid content [23]. Interesting bioactive properties are associated with fucoxanthin, which is a popular functional ingredient in food, cosmeceutical, and nutraceutical industries [24, 25]. However, the bioavailability of marine algae-derived bioactive small molecules like fucoxanthin or phlorotannins has not yet been sufficiently researched, especially in *S. fulvellum*. In a recent study [6], the grasshopper ketone (GK) (Figure 2(b)) was isolated and purified from the *n*-hexane fraction from *S. fulvellum*. Its structure was elucidated on the basis of spectral and chemical evidence and demonstrated that GK has anti-inflammatory activity and contributes to the treatment of inflammatory diseases. Ina et al. previously isolated an analog to chlorophyll-related compounds, pheophytin A (Figure 2(c)), from *S. fulvellum* and demonstrated that it is a neurodifferentiation compound [26]. According to Wu et al., two bioactive products identified as 1-O-palmitoyl-2-O-oleoyl-3-O-(*α*-D-glucopyranosyl)-lycerol and 1-O-myristoyl-2-O-oleoyl-3-O-(*α*-D-glucopyranosyl)-glycerol (Figure 2(d)) obtained from *S. fulvellum* showed fibrinolytic activity in the reaction system of single-chain urokinase-type plasminogen activator (pro-u-PA) and plasminogen [27].

*S. fulvellum* is known to produce different polysaccharides, like fucoidans (Figure 2(e)), alginates (Figure 2(f)), and laminarans (Figure 2(g)). Laminarans and fucoidans are the main water-soluble polysaccharides in brown algae, whereas high-molecular mass alginic acids represent alkali-soluble polysaccharides. Alginic acid has two basic components, *α*-1,4-linked L-guluronic acid and *β*-1,4-linked D-mannuronic acid (both of which are hexuronic acids), arranged in homopolymeric blocks separated by regions of alternating sequences of the two monomers [28]. Fucoidans are a class of sulfated polysaccharides commonly present in the extracts of brown seaweeds [29]. Fucoidan is largely composed of _L_-fucose and sulfate groups. It is used worldwide, especially by the food and pharmaceutical industries because of its diverse bioactive effects like anticoagulant, antioxidant, anticancer, anti-inflammatory, and immune-modulatory activities. Fucoidan is a homofucose polysaccharide usually classified into the following two types: the first (I) is composed of repeated (1 → 3)-_L_-fucopyranose units, while the other type (II) alternates repeated (1 → 3)- and (1 → 4)-_L_-fucopyranose units. Laminaran is a major storage polysaccharide present in brown seaweeds [30]. It represents 35% of the dry weight of seaweeds. Laminaran consists of glucose monomers joined together mainly by a (1,3)-*β*-D-glucan backbone with *β* (1,6) glycosidic bonds. In recent years, brown seaweeds have become important sources of natural compounds [31–33], many of which have been proven to possess bioactive effects [19, 34]. Therefore, the need to isolate novel bioactive compounds from edible seaweeds has increased recently.

## 4. Biological Activities of *S. fulvellum*

### 4.1. Antioxidant Activity

Oxidative stress has been associated with a number of lifestyle-related diseases, such as aging, arthritis, atherosclerosis, emphysema, cancer, and diabetes [35]. Marine seaweeds have been considered one of the main sources of natural antioxidants. Antioxidant properties *in vivo* and *in vitro* were tested in different ways, including free radical scavenging, lipid peroxidation inhibition, and the enhancement of superoxide dismutase (SOD), catalase (CAT), and glutathione peroxidase (GSH-Px) activities [36]. An antioxidant evaluation was performed on fucoidan (named SFSP, molecular weight (MW) = 529 kDa) extracted from *S. fulvellum* harvested from Jeju City. SFSP was selected because it exhibited more potent activity than the commercially available antioxidants, butylated hydroxyanisole and *α*-tocopherol. Results showed a dose-dependent hydrogen peroxide scavenging activity in the V79-4 cell line and a promising *α*, *α*-diphenyl-*β*-picrylhydrazyl (DPPH) free radical scavenging activity when compared to the commercial fucoidans (*Fucus vesiculosus and Undaria pinnatifida*) [7]. These fucoidans contained a significant amount of phenolic compounds, which suggested that the antioxidant activities of fucoidan could be attributed to the combined effect of polysaccharides and phenolic compounds. Interestingly, a study on the antioxidant activity of *S. fulvellum* methanolic extract (named SFME) demonstrated that it can strongly scavenge reactive oxygen species (ROS) such as superoxide anions, hydroxyl radicals, hydrogen peroxide, and DPPH free radicals. In addition, SFME also showed higher polyphenolic content (7.56 mg/g), which was correlated with superoxide anion and DPPH free radical scavenging abilities [37]. These results indicate that further investigations are needed to identify and purify the responsible antioxidative components. Wang et al. [36] investigated the antioxidant properties of polysaccharides in *S. fulvellum* extracts prepared using Celluclast (SFC) and found that SFCs scavenged DPPH, alkyl, and hydroxyl radicals, especially the alkyl radical (IC_50_ = 0.45 ± 0.11 mg/mL). Additionally, SFCs significantly reduced intracellular ROS levels and improved the viability of 2,2-azobis(2-amidinopropane) hydrochloride- (AAPH-) induced Vero cells in a dose-dependent manner. To date, the relationships between the antioxidant activity, structural features, and chemical structure of various polysaccharides are still unclear. Fortunately, various reports have indicated that the antioxidant activities of polysaccharides may be correlated with intrinsic viscosity and MW [38, 39], as well as monosaccharide composition, chemical structures, uronic acid content, and chain conformations [40]. Future studies also need to investigate the mechanisms underlying *S. fulvellum* antioxidant activity. They should also aim to improve antioxidant inhibition assays, which will be helpful for understanding the relationship between the chemical structures and antioxidant properties. The summarized antioxidant activities of the biologically active components of *S. fulvellum* provide a comprehensive evaluation of its value as a natural antioxidant source (Table 1).

### 4.2. Neuroprotective Activity

With the increase in life expectancy, normal brain aging and neurological disorders due to pathological degeneration of neurons are a growing concern for the elderly. The pathogenesis of neurological disorders tends to be associated with region-specific neuronal degeneration and atrophy, which is primarily caused by the reduced level of neurotrophic factors [41, 42]. The development of effective neuroprotective agents is important for the treatment of both acute and chronic brain disorders, such as traumatic brain injury, stroke, and Alzheimer's and Huntington's diseases [43, 44]. Seaweeds are potential sources for such agents [45]. So far, three compounds with neuritogenic activity have been identified from the genus *Sargassum*. Sargaquinoic acid and sargachromenol from *Sargassum macrocarpum* were found to promote neurite outgrowth in rat pheochromocytoma (PC12) cells in the presence of nerve growth factor at low concentrations [22, 46]. Another neurodifferentiation compound, pheophytin A (Figure 1(c)), identified and characterized from *S. fulvellum*, was shown to have a similar neuritogenic activity in PC12 cells [26]. This study suggested that pheophytin A at a concentration of 3.9 *μ*g/mL synergizes with nerve growth factor (NGF) to promote neurite outgrowth in rat pheochromocytoma PC12 cells via mitogen-activated protein kinase signaling. Moreover, in the follow-up study, the same group investigated the effects of the pheophytin A analog vitamin B_12_ on PC12 cell differentiation. Vitamin B_12_ demonstrated neurite outgrowth-promoting activity in PC12 cells, and in a manner similar to NGF, it stimulates the mitogen-activated protein kinase (MAPK) signaling pathway via the activation of extracellular signal-regulated kinase (ERK) [47]. However, it should be noted that PC12 cells are neurosecretory cells that originate from the peripheral nervous system and that they do not form neurites *in vivo*. Another point to note is that these compounds do not have the potential to initiate neuritogenesis by themselves; instead, they can enhance the neurite outgrowth of PC12 cells in the presence of low concentrations of nerve growth factor, but further efforts are required to evaluate the potential neurotrophic effects of *S. fulvellum* on neurons from the central nervous system (CNS). Hannan et al. [45] investigated the ethanol extracts of 22 seaweed species, including *Gelidium amansii*, *Undaria pinnatifida*, and *S. fulvellum* for their ability to protect mature neurons from atrophy in neurodegenerative diseases. In this preliminary screening experiment, the ethanol extracts of *S. fulvellum* (SFE) showed neurite outgrowth-promoting ability, thus helping the development of rat hippocampal neurons. In another study, the authors extended the previously reported neurite outgrowth-promoting activity of *S. fulvellum* in the PC12 cells to the CNS neurons and further proved its efficacy with respect to the formation of neural networks by enhancing synaptogenesis. Treatment of rat hippocampal neurons with SFE (5 *μ*g/mL) resulted in a significant enhancement of desirable effects, such as neuronal maturation in the early stage of development, cytoarchitecture complexity, and synaptogenesis in the later maturation stage. In addition to its effect on neurite development, SFE significantly increased the number of postsynaptic density-95 and synaptic vesicle 2 puncta and synapses (about 35%, 67%, and 125%, respectively, with respect to that in the control) [12]. Notably, small molecules isolated from *S. fulvellum* showed higher protective effect against neurite damage in cases of excessive neuronal cell loss, indicating that *S. fulvellum* can promote neuronal maturation and synaptogenesis, and support neuronal survival. Thus, *S. fulvellum* could have potential therapeutic value for brain aging as well as for neurodegenerative diseases by favoring the reconstruction of the partially damaged neuronal network.

### 4.3. Immunoinflammatory Activity

Seaweeds produce bioactive compounds, including fucoidans, fucosterol, and phlorotannins, which are responsible for regulating immune-signaling and anti-inflammatory activity [48–52]. *S. fulvellum* has been shown to possess immunomodulatory properties that are potentially applicable for stimulating the immune response or controlling immune cell activity for mitigating associated negative effects such as inflammation [53]. These marine sources of medicinal compounds may also affect multiple targets in the immune and inflammatory systems that are important for disease progression. The immunomodulatory activities of a *S. fulvellum* polysaccharide (SFP) have been investigated previously in RAW 264.7 macrophages and mouse splenocytes *in vitro* by Sung et al. [13]. It was found that SFP induced macrophage activation by increasing the expression of CD80 and CD86 molecules, and it resulted in the production of markedly higher levels of proinflammatory cytokines (tumor necrosis factor- (TNF-) *α*, interleukin- (IL-) 6, and IL-12p70) through the activation of nuclear factor kappa B (NF-*κ*B), MAPK signaling, and the T helper 1 (Th1) immune response. Similarly, Byun [54] compared the immunomodulatory effects of SFP with those of SFE in macrophages and murine splenocytes and confirmed that the immunomodulation may be dependent on polysaccharide extracts. This study demonstrated that SFP significantly increases nitric oxide (NO) and cytokine production (TNF-*α*, IL-6, and IL-1*β*), whereas SFE does not cause increased NO and cytokine production. In the case of splenocytes, SFP increased splenocyte proliferation and Th1 type cytokine (IL-2 and interferon- (IFN-) *γ*) production to a greater extent than SFE. The above results suggested that the immunomodulatory activity of SFP was mainly mediated via the regulation of Th1-related cytokine expression. The time-effect relation of the cytokine response also indicated that macrophages and natural killer cells involved in nonspecific immunity were primarily activated and that helper T cells were secondarily affected in response to SFP treatment.

Direct stimulation of immune cells by *S. fulvellum* extracts results in the production of NO through the induction of inducible nitric oxide synthase (iNOS) and in the generation of a proinflammatory cytokine/chemokine profile. Depending on the situation, the interaction of *S. fulvellum* extracts with other effectors may result in reduced inflammation. For example, NO inhibition was highly increased in lipopolysaccharide- (LPS-) treated macrophages in the presence of *S. fulvellum* aqueous extracts. Studies also showed that *S. fulvellum* extracts increased RAW 264.7 macrophage proliferation by more than 80% [53]. Jeong et al. [55] confirmed that NO and TNF-*α* secretion were significantly inhibited when LPS-stimulated RAW 264.7 cells were treated with *S. fulvellum* water extracts (SFWE). Moreover, SFWE inhibited the expression of IL-6 and IL-1*β* in a dose-dependent manner. In particular, IL-6 inhibition activity was >50% at a concentration of 1% SFWE. Jaswir et al. [56] showed that *S. fulvellum* ethanol extract (SFEE) inhibited not only the production of NO and proinflammatory cytokines (IL-6, IL-1*β*, and TNF-*α*) but also the expression of iNOS and cyclooxygenase 2 in LPS-stimulated RAW 264.7 cells without affecting their viability. SFEE also suppressed the expression of NF-*κ*B, suggesting that SFEE could affect the expression of inflammation-related cytokines and proteins through the regulation of NF-*κ*B. Furthermore, the formation of ear edema was 40% lower in mice treated with the highest dose (250 mg/kg) of SFEE than that in the control mice [57].

There is cumulative evidence to suggest that *S. fulvellum* extracts have anti-inflammation potential that modulate pro-/anti-inflammatory cytokine secretion profiles. Gwon et al. [10] investigated the inhibitory effect of hexane extract from *S. fulvellum* on the LPS-stimulated RAW 264.7 cells and phorbol 12-myristate 13-acetate- (PMA-) induced mouse-ear edema. The hexane fraction of *S. fulvellum* (HFS) inhibits the production of proinflammatory mediators and cytokines. Moreover, the anti-inflammatory effects of HFS on LPS-treated cells are associated with the inactivation of the NF-*κ*B pathway implicated with the suppression of NF-*κ*B through the inhibition of MAPKs and Akt (cellular homolog of murine thymoma virus *akt8* oncogene) phosphorylation. Verification of HFS anti-inflammatory activity and relative mechanism at the cellular and molecular levels will be beneficial for its application in therapeutic agents for treating inflammation-mediated diseases. Recently, the grasshopper ketone (GK) (Figure 1(b)) extracted from *S. fulvellum* showed anti-inflammatory activities in LPS-induced RAW 264.7 murine macrophage cell line. The production of proinflammatory cytokines (IL-6, IL-1*β*, and TNF-*α*) was significantly reduced in 0.1-100 *μ*g/mL dose ranges of GK treatment. Furthermore, the dose-dependent and significant inhibition of iNOS and cyclooxygenase-2 (COX-2) protein expression was confirmed. The GK exerts its anti-inflammatory effect by inhibiting MAPKs (ERK, c-Jun-N-terminal kinase (JNK), and p38 kinase) and NF-*κ*B p65 phosphorylation [6]. In another study, GK isolated from SFEE had an inhibitory effect on atopic dermatitis (AD) on 2,4-dinitrochlorobenzene- (DNCB-) induced AD-like skin lesions in BALB/c mice. In this study, SFEE inhibited the development of AD-like skin lesions and decreases serum IgE and cytokine levels (IL-4, IL-5, IL-10, IL-13, IFN-*γ*, and TNF-*α*), while the GK suppressed the expression of IFN-*γ* and IL-4 in mouse splenocytes [58]. Overall, this study demonstrated that the anti-inflammatory properties of GK on macrophages were achieved by inhibiting the NF-*κ*B and MAPK pathways, which are associated with the attenuation of cytokine secretion [6].

As is well known, UV radiation is an important etiologic factor for inflammatory skin damages, oxidative stress, DNA damage, cellular and tissue injuries, cell death, skin cancer, and premature skin aging [59–61]. In a study performed by Lee et al. [62], the experimental HaCaT keratinocytes and BALB/c mice were treated with the ethyl acetate fraction of *S. fulvellum* ethanol extract (SFE-EtOAc); it was found that SFE-EtOAc could be an effective anti-inflammatory agent protecting against UVB irradiation-induced skin damage. The results showed that SFE-EtOAc effectively inhibited UVB-induced cytotoxicity and the production of proinflammatory proteins or mediators including COX-2, TNF-*α*, iNOS, prostaglandin (PG) E2), and NO, both in *in vitro* HaCaT human keratinocytes and in *in vivo* BALB/c mice.

These and other studies report of bioactive compounds derived from *S. fulvellum* with immunomodulatory and anti-inflammatory properties. However, the effect of *S. fulvellum* on immunoinflammatory activities and its underlying molecular mechanisms have not been studied and remain largely unresolved. Further studies are required to determine the anti-inflammatory potential of *S. fulvellum* extracts and their application in food and pharmaceutical products. These extracts should be further purified and characterized to possibly identify the relationship between their anti-inflammatory role and chemical structure, which would provide a novel insight into the application of *S. fulvellum* in the development of alternative anti-inflammatory drugs.

### 4.4. Anticoagulant and Antithrombotic Effects

Anticoagulants inhibit or decrease the ability of blood to clot or coagulate and therefore help preventing the formation of harmful clots in blood vessels [63]. Heparin is a highly sulfated polysaccharide present in mammalian tissues and a widely used anticoagulant for the treatment and prevention of thrombotic diseases [64]. Alternative drugs for heparin are in high demand, due to its adverse effects, such as occasional life-threatening bleeding and thrombocytopenia. Thus, the disadvantages associated with heparin motivate further research on novel substances with anticoagulant properties [35]. Thus, anticoagulant and antithrombotic activities are the most studied properties of sulfated polysaccharides from seaweeds [65–67].

The compounds and extracts of *S. fulvellum* also possess anticoagulant and antithrombotic effects, and it was thought that sulfated polysaccharides were responsible for this [68]. De Zoysa et al. [15] found that the anticoagulant activity of fermented *S. fulvellum* sulfated polysaccharides was closely related to the inhibition of both intrinsic and extrinsic pathways of coagulation, but not to the inhibition of thrombin activity or fibrin polymerization. Freeze-dried *S. fulvellum* was fermented in an incubator for 10 weeks at 25°C to convert seaweed macromolecules into anticoagulant sulfated polysaccharides (ASP). The anticoagulant activity of ASP was measured using activated partial thromboplastin (APTT), thrombin time (TT), and prothrombin (PT) clotting time assays. The results suggested that both ASP and heparin showed a relative clotting factor of 27.47 at the concentrations of 180 and 60 *μ*g/mL, respectively. Chemical characterization further showed that *S. fulvellum* ASP had a considerable amount of negatively charged sulfate, which may be able to interact with coagulation factors and thus initiate the coagulation inhibition [15]. In another study, Jo and Choi [69] investigated the P-selectin binding and the antithrombotic and hemolytic activity of the depolymerized fucoidans extracted from *S. fulvellum*. The fucoidans degraded by ultrasound (US) or electron beam (EB) irradiation in the presence of hydrogen peroxide aqueous solution and the low molecular weight fucoidans (LMWFs) prevented P-selectin binding to Sialyl Lewis X with an IC_50_ (inhibitory concentration 50) of 20 nM as compared to heparin (400 nM) and dextran sulfate (25,000 nM). This means that LMWFs in this study were much more effective in antithrombotic activity than heparin and dextran sulfate. Notably, the LMWFs showed no hemolytic activity at concentrations of up to 950 *μ*g/mL. Thus, it might be justified to state that LMWF is safer than heparin and its constituents in clinical use. These results showed that *S. fulvellum* sulfated polysaccharide (fucoidan) has high blood anticoagulation activity and significantly prolongs the time preceding thrombosis and shortens thrombosis duration.

### 4.5. Hepatoprotective Activity

Despite the huge advancements in modern medicine, drugs able to effectively stimulate hepatic function offer complete protection to the liver, or aid in the regeneration of hepatic cells is yet undiscovered. Additionally, some drugs also induce adverse or side effects. Thus, it is necessary to develop alternative pharmaceuticals for the treatment of hepatic diseases, based on more effective and less toxic agents [70, 71]. Kawano et al. [72] reported that diets containing edible seaweeds (*S. fulvellum*) prevented liver toxicity induced by D-galactosamine in rats. It also normalized the elevated levels of serum alanine transferase (ALT) and those of GSH in the cecum. However, they have not clarified the components or factors in *S. fulvellum* able to repress D-GalN-hepatopathy [73]. In a following study, the authors suggested that the protective effect of the three kinds of *S. fulvellum* against D-GalN-hepatopathy could be partly attributed to fucoidan. This study proposed one plausible mechanism, i.e., the marked increase of GSH levels in cecal contents as a result of repeated intake of *S. fulvellum* could promote the D-GalN-hepatopathy defense mechanism by influencing bacterial translocation to the intestine. The obtained results demonstrate the hepatoprotective activity of *S. fulvellum* fucoidan and provide new prospects for its clinical application in the treatment of hepatic diseases.

### 4.6. Other Biological Activities

Other biological effects include fibrinolytic, antipyretic, analgesic, antiatopic, antibacterial, anticancer/antitumor, and antidiabetic activities (Table 1). Two novel glucopyranosyl diacylglycerols (Figure 1(d)) isolated from *S. fulvellum* with a backbone composed of 1-O-palmitoyl-2-O-oleoyl-3-O-(*α*-D-glucopyranosyl)-glycerol (POGG) and 1-O-myristoyl-2-O-oleoyl-3-O-(*α*-D-glucopyranosyl)-glycerol (MOGG) were shown to exhibit fibrinolytic activity in a reaction system comprising pro-u-PA and plasminogen [27]. Kang et al. [11] revealed that tetradecanoic acid, hexadecanoic acid, neophytadiene, and oleic acid from *S. fulvellum* show potent antipyretic, analgesic, and anti-inflammatory activities, especially antiedema activity. Yoon et al. [74] reported that the ethanol extract from *S. fulvellum* (SFE) showed antibacterial activity and that 70% SFE significantly inhibited the *α*-glucosidase activity *in vitro*, with an IC_50_ value of 8.13 *μ*g/mL [75]. Another recent report revealed that the *α*-glucosidase inhibitory activity can be enhanced using *S. fulvellum* enzymatic extracts, which also exhibited potent antioxidant and alcohol dehydrogenase activities [76]. Additionally, Jo et al. [77] reported the antitumoral effects of *S. fulvellum* hot-water extract and elucidated the potential mechanisms using both *in vitro* and *in vivo* systems. They found that the modulation of the Bcl-2 family members and activation of the p53 pathway may be closely related to the prevention of cancer and may therefore function as an important antitumoral mechanism.

## 5. Conclusion and Future Perspectives

*S. fulvellum* is a popular and inexpensive edible brown seaweed that is rich in nutrients (carbohydrates, unsaturated fatty acids, protein composition, vitamins, and minerals) and in natural bioactive compounds, such as phlorotannins, grasshopper ketones, and polysaccharides. Over the past twenty years, phytochemical investigations have led to the isolation of a large number of bioactive SFPs and other purified fractions. Bioactive low-molecule compounds (phlorotannins, fucoxanthin, astaxanthin, canthaxanthin, peridinin, fucoxanthinol, etc.) and polysaccharides are considered the major constituents of *S. fulvellum* that show many pharmacological effects, including antioxidant, immunomodulatory, anti-inflammatory, hepatoprotective, anticoagulant, and neuroprotective activities. However, systemic data are still insufficient regarding the pharmacokinetics and toxicity of this seaweed, especially target-organ toxicity; therefore, more investigations should be performed in the future. Furthermore, the biological activities of polysaccharides are closely related to their rheological properties, MW, chemical structures, constituent monosaccharides, substituent groups, and chain conformations [78]; thus, in-depth structural analysis of SFP needs to be systematically performed to assess the exact chemical structures of SFP.

More importantly, some issues still need to be taken into consideration; these include (1) the extraction method used for SFP preparation, which is currently exclusively hot-water extraction; (2) the lack of uniformity in raw materials and extraction and purification methods, implying SFP with differences in structure and biological activities; (3) the effects on human tissues that need to be studied, as *in vitro* cell experiments cannot completely reflect the complexity of a real organism-level response; (4) unclear bioactive components which are responsible for each bioactivity; (5) the fact that the development of the physiological activity of *S. fulvellum* extracts also presents its own limitations; therefore, future developments with respect to novel SFP extraction techniques (such as ultrasound, ultrahigh pressure, and multitechnology combination) are needed. Standard separation and purification methods also need to be established to obtain a homogeneous product, i.e., polysaccharides with high purity, high bioactivity, and high reproducibility. Moreover, in order to better determine the authenticity of *S. fulvellum* extracts on human health, *in vivo* experiments and clinical studies must be performed. Furthermore, in order to employ *S. fulvellum* for a wider range of applications, we recommend prompt screening and isolation to develop novel functional compounds from this seaweed.

## Figures and Tables

**Table 1:** Summary of the biological activities of the brown seaweed *S. fulvellum*.

Therapeutic activity	Extract/compound	Pathway mode/effect	References
Antioxidant	Fucoidan	DPPH, HO^·^, NO^·^ radical scavenging Hydrogen peroxide scavenging	[7]
	Enzymatic extract	DPPH radical scavenging	[76]
	Enzyme-assistant extracts	DPPH, alkyl, and hydroxyl radical scavenging Reduced intracellular ROS levels	[36]

Neuroprotective	Pheophytin A	Activation of a MAPK signaling pathway	[26]
	Ethanol extract	Promoted the initial neuronal differentiation and increased the indices of axonal and dendritic development	[45]

Anti-inflammatory	Ethanol extract	Suppressed production of NO, IL-6, TNF-*α*, and IL-1*β*	[79]
	Water extract	Suppressed the NO and TNF-*α* secretion	[55]
	Seaweed supplementation	Reduced circulation of IL-1*β* and IL-6	[80]
	Grasshopper ketone	Inhibiting MAPKs (ERK, JNK, and p38) and NF-*κ*B p65 phosphorylation	[6]
	Aqueous extract	Inhibited NO production	[56]
	Water extract	Inhibited NO production	[53]
	Hexane fraction	Downregulation of NF-*κ*B activity via the inhibition of MAPKs and Akt phosphorylation	[10]
	Grasshopper ketone	Regulatory effects of IL-4, IL-5, IL-10, IL-13, IFN-*γ*, and TNF-*α*	[58]
	Hot-water extract	Inhibited the expression levels of IL-1*β*, IL-4, IL-5, IL-6, IL-8, and IL-13 via NF-*κ*B Activated ERK/P38	[48]
	Ethanolic extract	Suppressed NF-*κ*B activation	[57]
	Ethanolic extract	Inhibited the expression of proinflammatory proteins such as COX-2, TNF-*α*, and iNOS	[62]
	Water extract	Modulation of IL-4 and IFN-*γ*	[81]

Anticoagulative	Enzymatic extract	Inhibited the serine proteases II, X, and VII	[68]
	Fucoidan	Inhibited both intrinsic and extrinsic blood coagulation pathways	[15]

Antidiabetic	Seaweed supplementation	Reduced insulin resistance	[80]
	Enzymatic extract	*α*-Glucosidase inhibitory	[76]
	Ethanolic extract	*α*-Glucosidase inhibitory	[75]

Immunomodulatory	Polysaccharides	Via activation of NF-*κ*B and MAPK signaling and the Th1 immune response	[13]
	Polysaccharides and ethanol extract	Increased splenocyte proliferation and production of Th1-type cytokines	[54]
	Grasshopper ketone	Inhibition of T cell activation	[58]

Hepatoprotective	Seaweed supplementation	Increase of GSH level in cecal contents	[73]
	Fucoidan	Through intestinal bacteria fermentation, but further study is necessary to completely clarify	[72]

Anticancer	Hot-water extract	Upregulation of p53	[77]
	Sodium alginate	Inhibited uptake of ^3^H-thymidine	[82]
	Polysaccharide	Inhibited the growth of subcutaneous sarcoma-180	[14]
	Hot-water extract	Inhibited the growth of subcutaneous sarcoma-180	[83]
	Organic solvent extract	High cytotoxicity to HepG2, HT⁃29, and HeLa	[77]

Fibrinolytic	Glucopyranosyl diacylglycerols	Enhanced reciprocal activation of pro-u-PA and plasminogen	[27]

Antimicrobial	Ethanol extract	Antibacterial	[74]

Antithrombotic	Fucoidan	Prevented P-selectin binding	[69]

## Additional Points

*Summary of the Literature*. This review is aimed at presenting highlights from literature to provide novel insights into the potential of *Sargassum fulvellum* and its active compounds for use as innovative therapies.  Section 1, which is Introduction, provides an overview of the general properties of *S. fulvellum.*Section 3 focuses on the key biologically active components of *S. fulvellum*, including the structure of *S*. *fulvellum* polysaccharides, their characterization, and composition; all these compounds have the potential to be developed as components of functional foods. Finally, Section 4 analyzes past studies on the pharmacological properties of *S. fulvellum*, which were divided into antioxidant, neuroprotective, immune-inflammatory, anticoagulative, and hepatoprotective; in each subsection, we focused on our increasing comprehension, highlighting the benefits of this macroalgae in human disease treatment. The present review discusses the phytochemical, pharmacological, therapeutic, nutritional, and health benefits of *S*. *fulvellum*, as well as its use in the prevention of diseases and maintenance of good health.
